# General Method to Synthesize Highly Stable Nanoclusters via Pickering-Stabilized Microemulsions

**DOI:** 10.1021/acs.langmuir.3c00221

**Published:** 2023-04-18

**Authors:** Wei Zou, Cui Wang, Jiasheng Wang, Jia Xiang, Götz Veser, Shufen Zhang, Rongwen Lu

**Affiliations:** † State Key Laboratory of Fine Chemicals, Frontiers Science Center for Smart Materials Oriented Chemical Engineering, Dalian University of Technology, Dalian 116024, P. R. China; ‡ Department of Chemical Engineering, University of Pittsburgh, Pittsburgh, Pennsylvania 15261, United States

## Abstract

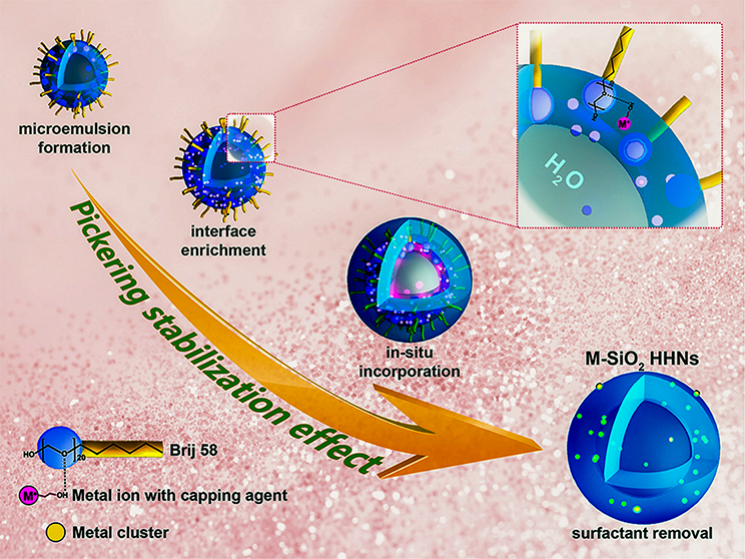

The ability to not only control but also maintain the well-defined size of nanoclusters is key to a scientific understanding as well as their practical application. Here, we report a synthetic protocol to prepare and stabilize nanoclusters of different metals and even metal salts. The approach builds on a Pickering stabilization effect inside a microemulsion system. We prove that the emulsion interface plays a critical role in the formation of nanoclusters, which are encapsulated in situ into a silica matrix. The resulting nanocapsule is characterized by a central cavity and a porous shell composed of a matrix of both silica and nanoclusters. This structure endows the nanoclusters simultaneously with high thermal stability, good biocompatibility, and excellent photostability, making them well suited for fundamental studies and practical applications ranging from materials chemistry, catalysis, and optics to bioimaging.

## Introduction

Metal nanoclusters in the size regime below 2 nm represent a class of materials with unique physical and chemical properties, often considered a bridge between isolated atoms and bulk materials.^1−5^ Insights into the physics and chemistry of nanoclusters hence not only advance our fundamental understanding of materials science but also enable utilization of their unique properties in a wide range of potential applications.^6−9^ For example, the active components of the most efficient heterogeneous catalysts are often very small metal nanoclusters.^10−14^ Similarly, the size-dependent fluorescent emission of metal nanoclusters upon photoexcitation in the UV–visible range^15,16^ is critical for applications in biological imaging and nanoscale optoelectronics.^17−19^

However, nanoclusters are intrinsically unstable due to their very small size. The large portion of surface atoms with unsaturated coordination makes nanoclusters prone to aggregation and sintering.^20^ For example, Pd nanoclusters are known to start sintering at temperatures as low as ∼200 °C.^21^ Hence, metal nanoclusters readily grow into larger particles and thus lose their functionality when they are used at technical conditions.^22^ Recent progress in the controlled synthesis of metal nanoclusters has witnessed the successful application of suitable capping agents for the stabilization of nanoclusters.^20^ These agents deactivate the nanocluster surface so that they remain well dispersed in solution, resulting in a longer stock life. However, even these water-soluble nanoclusters, such as glutathione-capped Au nanoclusters, will tend to aggregate at elevated temperatures.^23^ Moreover, the dissolution of nanoclusters required for this stabilization makes them hard to handle and recycle in practical usage. For systematic investigations of the unique properties of nanoclusters and to fully enable their technical potential, synthetic protocols are needed which are effective for a broad variety of nanoclusters. It is furthermore desirable that these protocols result in sufficient quantity and stability of nanoclusters and yield them in a form in which they can be easily collected and processed for further investigation or application.

We have previously reported the controlled synthesis of larger nanoparticles with special attention to their thermal stability.^23^ In a continuation of this effort, we report here an advance in the design and synthesis of stabilized nanoclusters. The synthetic protocol builds on the formation of a so-called “Pickering emulsion”, a thermodynamically stable emulsion in which colloid particles act as effective stabilizers of the emulsion interface.^24^ Instead of utilizing colloidal particles to stabilize emulsion droplets via the Pickering stabilization effect,^25^ we reversed the approach by exploring the possibility of using a preformed emulsion interface to stabilize solid species, i.e., specifically stabilize otherwise unstable nanoclusters. Following this direction, we successfully developed a new methodology to prepare metal nanoclusters inside a water-in-oil (W/O) microemulsion, including, but not limited to, Ru, Co, Ni, Pd, Pt, Cu, Ag, Au, and Pb. Furthermore, we were also able to expand our synthesis to nonmetallic nanoclusters such as hydroxides of Fe^3+^ and even metal salts (e.g., (NH_4_)_6_Mo_7_O_24_ or (NH_4_)_6_W_7_O_24_). The final product, M–SiO_2_ (M = different metals or metal salts), shows a unique hybrid hollow nanosphere (HHN) structure characterized by a central cavity and a composite wall composed of nanoclusters embedded in porous silica. The existence of a thin ceramic SiO_2_ matrix endows the composite with a number of desirable characteristics for application, such as good biocompatibility, processability, and outstanding stability.

## Experimental Section

**Scheme 1:**
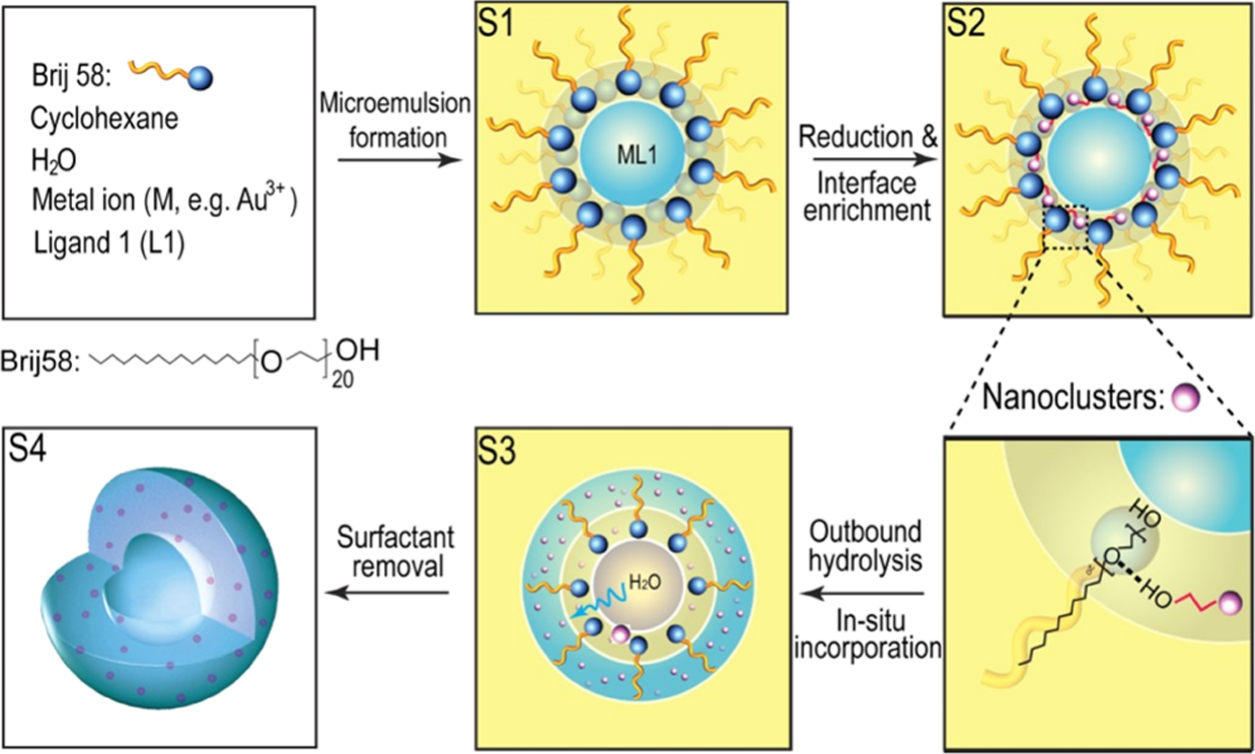
Proposed Mechanism for the Formation of M–SiO_2_ HHNs Metal ions (e.g., Au^3+^) are coordinated with ligand 1 (L1) inside the water droplets of the microemulsion (Stage 1, S1). Fast reduction results in the formation of nanoclusters (pink spheres), which get enriched at the emulsion interface (S2; the magnified image details the interface interaction). The hydrolysis of TEOS induces outbound diffusion of water and, thus, the formation of a silica shell (blue) around a central cavity (S3). Nanoclusters diffuse out with the water and are incorporated into the silica matrix. Removal of organic residues results in the formation of hollow nanospheres with silica-embedded nanoclusters as the final morphology (S4).

A typical synthesis starts with metal salts dissolved in a regular microemulsion system (Scheme 1). We take the synthesis of Au–SiO_2_ HHNs as an example. A clear W/O microemulsion is formed by using Brij 58 as the nonionic surfactant. *N*-(2-hydroxyethyl)ethylenediamine is used as stabilizing agent (labeled as L1), introduced to coordinate with Au^3+^ to avoid precipitation in the microemulsion (Stage 1, S1). A suitable reducing agent, e.g., NaBH_4_ for Au^3+^, is used to trigger a fast reduction during which very small metallic Au species form. To prevent the newly formed Au from growing into larger particles, mercaptoethanol is used to passivate the particle growth due to the strong Au–S bond.^26^ As discussed in detail further below, metallic Au is first formed inside the water droplets and then attaches to the W/O interface (S2). The resulting enrichment of the micellar interface with Au nanoclusters results in a Pickering stabilization effect and changes the properties of the emulsion interface, resulting in an outbound diffusion of water during the following hydrolysis upon the slow addition of tetraethyl orthosilicate (TEOS) to the reaction system (S2). Nanoclusters on the interface are carried out and embedded into the newly formed silica shell (S3). Finally, depletion of the water droplets leaves an empty cavity in the center. The formed Au–SiO_2_ HHNs can be easily collected via washing and centrifugation cycles and then dried in a conventional oven for further characterization and use (S4).

## Results and Discussion

**Figure 1:**
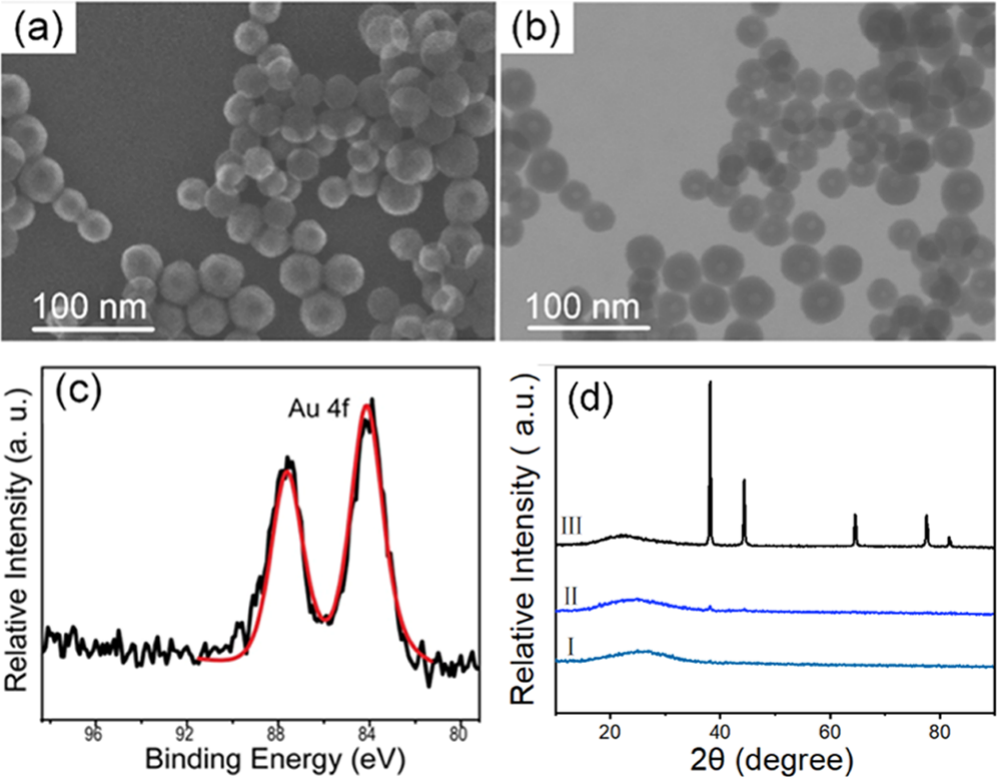
Characterization of a typical Au–SiO_2_ HHN sample. (a) SEM image. (b) TEM image. (c) XPS spectrum. (d) XRD pattern for the fresh Au–SiO_2_ HHNs (I), pure Au nanoclusters (II), and Au–SiO_2_ HHN samples after heating to 600 °C (III).

Figure 1a shows a typical scanning electron microscopy (SEM) image for a collected Au–SiO_2_ HHN sample. The powder consists of uniform nanospheres with a diameter of 39 ± 4 nm. Transmission electron microscopy (TEM) (Figure 1b) reveals that these nanospheres have a hollow center, unlike solid silica particles obtained from a regular TEOS hydrolysis process.^27^ In agreement with the SEM result, the particle size of these nanospheres is around 40 nm, with a central cavity of about 15 nm. X-ray photoelectron spectroscopy (XPS) is used to probe the state of Au in the powder. As shown in Figure 1c, the Au 4f_7/2_ XPS spectrum exhibits characteristic doublet peaks at 84.3 and 87.8 eV, indicating the existence of metallic Au. No signals for ionic Au can be detected, i.e., all Au species appear to be metallic in the final sample. Meanwhile, X-ray diffraction (XRD) characterization only shows a peak between ∼25–30° (Figure 1d, curve I), typical for amorphous SiO_2_.^28^ No characteristic peaks for metallic Au can be found, suggesting that the existing Au species are in a very small size range (<2 nm).^29^ Since the silica shell makes resolution of the Au nanoclusters in TEM difficult, a sample is collected before the introduction of TEOS and subjected to TEM investigation. Figure SI1 shows the presence of Au nanoclusters with a diameter below 1 nm (approximately ∼0.8 nm). These nanoclusters also do not show obvious XRD reflections of Au (Figure 1d, curve II). For the Au–SiO_2_ HHNs sample, a detailed TEM investigation confirms the existence of only one shape, namely, the hollow nanospheres, with no separate Au nanoclusters. This suggests that the Au nanoclusters and silica must coexist in these nanospheres, i.e., Au is highly dispersed in the shells of the formed hollow structures.

Generally, high-resolution TEM (HRTEM) is a well-established tool to investigate nanostructures. Unfortunately, the highly porous silica matrix of the thin nanoshells shows insufficient stability under the incident electron beam during HRTEM. Figure SI2 shows the morphology change of a selected area in TEM at a time interval of 30 s. To further confirm the presence of Au in these HHNs, we heated the sample to elevated temperatures in order to induce the formation of larger particles via sintering. Interestingly, the hollow nanosphere structure proved to be exceptionally stable against thermal degradation. The hollow nanospheres remain virtually unchanged after heating to temperatures as high as 500 °C. Finally, at temperatures of ∼600 °C, Au aggregates to larger nanoparticles with a diameter of around 6 nm inside the silica matrix (Figure SI3). XRD characterization confirms the formation of well-crystallized Au via the emergence of characteristic peaks for Au (Figure 1d, curve III, JCPDS #65-8601).^30^ The appearance of sintered Au nanoparticles inside silica hence further confirms the existence of Au nanoclusters inside the original shell and supports our identification of these composite nanostructures as hybrid hollow nanospheres (HHNs) with a shell composed of both Au nanoclusters and a porous silica matrix.

Nanoclusters of noble metals are known to exhibit unique optical properties, different from their larger counterparts.^8,31^ We therefore tested the optical properties of the Au–SiO_2_ HHN sample for a better understanding of these embedded Au nanoclusters. Figure S4a shows a typical UV–vis spectrum of the Au–SiO_2_ HHN powder redispersed in ethanol. No obvious peaks can be observed, with only a continuous decay typical for Au nanoclusters.^31^ For small clusters, Mie’s theory is not applicable, and their plasmon adsorption will disappear, resulting in the complete disappearance of UV–visible extinction bands. The Au–SiO_2_ HHN suspension shows a bright blue color when exposed to a UV lamp at 365 nm (Figure SI4c). Its corresponding fluorescent spectrum reveals a strong emission peak centered at around 376 nm (Figure SI4d). A series of experiments were carried out to identify the fluorophore in the HHN sample (Figure SI5a,b). First, the sample was heated to 400 °C to remove any organic residuals. The fact that the characteristic blue fluorescence remains unchanged demonstrates that organics are not its origin. Second, silica and Au in the heat-treated sample are etched separately via solutions of HF and aqua regia, respectively. The removal of silica by HF does not have an obvious effect on the fluorescence (Figure SI5b, center). In contrast, the use of an aqua regia solution to dissolve the metallic Au indeed results in complete quenching of the fluorescence. It is worth noticing that the loss of silica protection does affect the optical properties of Au nanoclusters in the long term. Due to the loss of the stabilizing silica shell during HF etching, Au nanoclusters aggregate gradually into larger particles (inset in Figure SI5b) and lose their characteristic fluorescence. Accordingly, the UV–visible spectrum shows a characteristic peak around 535 nm (Figure SI5c), which is typical for larger gold nanoparticles.^32^

**Figure 2:**
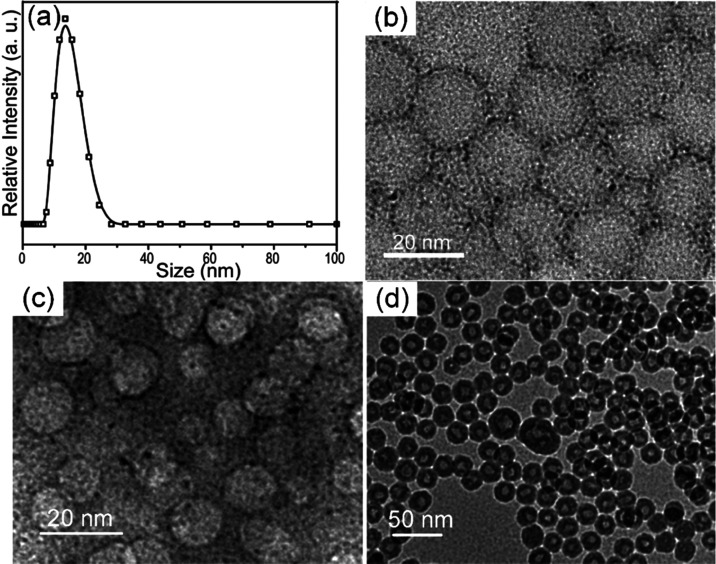
Structural evolution of Au–SiO_2_ HHNs. (a) DLS scan of the microemulsion. (b–d) TEM images of Au–SiO_2_ HHN samples collected at different reaction times after addition of tetraethyl orthosilicate: 30 min (b), 60 min (c), and 2 h (d).

Further experiments were conducted to identify the mechanism of formation for these HHN nanostructures. Dynamic light scattering (DLS) shows the micelle size of the microemulsion to be around 15 nm (Figure 2a), which is in good agreement with the cavity size of the HHNs. Time-dependent experiments are used to track the gradual formation of the hybrid wall. Samples after different periods of TEOS hydrolysis are collected and imaged in TEM. At the early stage of hydrolysis, the TEM examination shows the formation of thin spherical rings, as shown in Figure 2b. A hollow structure can be observed with tiny nanoclusters, presumably the preformed Au species, circling the surface. As the hydrolysis continues, more silica is formed, leading to a much clearer central cavity with improved phase contrast (Figure 2c). Finally, the continuous reaction produces uniform hollow nanospheres upon extended reaction time (Figure 2d). It should be noted that we did not observe an obvious change of the central cavity during the entire sol–gel process. Combined with the DLS data, it is evident that the hydrolysis starts right from the water–oil interface and then moves outward to form a solid shell around the central cavity. The hydrophilic Au nanoclusters, due to mercaptoethanol capping, will be carried simultaneously with the outward diffusion of water and then get embedded in situ during the formation of silica. For comparison, a traditional sol–gel process results in the formation of solid nanospheres under microemulsion conditions, as confirmed in a blank test in which solid nanospheres were formed under identical conditions when no HAuCl_4_ is added (Figure SI6a). Further experiments showed that a HAuCl_4_ concentration of 0.2–0.8 M is needed in the synthesis solution in order to assure the formation of Au–SiO_2_ HHNs.

A series of control experiments is used to demonstrate the role of key reagents in the synthesis of HHNs (Figure SI6). *N*-(2-hydroxyethyl)ethylenediamine is the coordinating agent (Ligand 1) used to stabilize HAuCl_4_ initially, while NaBH_4_ is necessary to induce a rapid reduction of the Au^3+^ so that a burst of a large number of nuclei yields very small metal particle sizes. The absence of NaBH_4_ produces only SiO_2_ solid nanospheres with no cavity (Figure SI6b). The exclusion of mercaptoethanol in the reaction system forms larger Au nanoparticles with no coexisting HHNs (Figure SI6c). After the reduction, the newly formed Au nuclei are very active and readily aggregate into larger particles. A strong capping agent with a mercapto-group is hence required to passivate the growth of Au.^33^ For comparison, other capping agents, e.g., ethanolamine, fail to constrain the growth of Au due to a weak bonding with the Au surface (Figure SI6d). However, the mercapto-group alone is not enough to guarantee the formation of Au nanoclusters. The coexistence of the strongly hydrophilic hydroxyl group also turns out to be indispensable to assure stable dispersion of the Au nanoclusters. For example, the use of mercaptoethane (Figure SI6e) or mercaptoacetate (Figure SI6f) produces large Au particles and a solid silica phase instead of the HHN structure.

In sum, our observations support that the formation processes of Au nanoclusters and the hollow nanospheres are interdependent. The emulsion interface is modified by the existence of solid nanoclusters, as revealed by the formation of hollow nanospheres. Larger particles, such as colloids, are known to be able to stabilize emulsion droplets by settling on the emulsion interface. For example, 10 nm solid capsules have been shown to form with 0.9 nm colloidal particles acting as surfactants at the interface.^34^ The decisive role that nanoclusters play in the formation of HHNs suggests that they function in a similar way to the role of colloids in the formation of microcapsules but on a much smaller length scale (nm vs μm). Hence, a Pickering stabilization effect is exerted by these interface-enriched nanoclusters (Figure 2b), producing a characteristic nanocapsule structure. To further probe the driving force for the enrichment of Au nanoclusters at the emulsion interface, Fourier transform infrared (FT-IR) characterization is carried out (Figure SI7). Upon stepwise addition of mercaptoethanol, the ν_C–O–C_ of Brij 58 at 1116 cm^–1^ shows a gradual shift toward a lower wavenumber, indicating the existence of a hydrogen bond between mercaptoethanol and the surfactant Brij 58.^35^ We therefore propose that nanoclusters capped by mercaptoethanol are caught and anchored at the emulsion interface through its interaction with Brij 58, preventing the self-aggregation of nanoclusters into larger particles that is observed in the absence of hydrogen bonding, as is the case for mercaptoethane. The emulsion interface thus forms an effective “docking area” for the stabilization of nanoclusters, and the hollow structure forms as a result of the Pickering stabilization based on hydroxyl group-terminated Au nanoclusters.

The successful synthesis of Au–SiO_2_ HHNs and our understanding of the mechanism underlying their formation inspired us to apply this synthetic protocol to further metals, especially those noble metals which can exhibit unique photonic, electronic, and catalytic properties. Based on the proposed mechanism, the selection of suitable stabilizing reagents becomes the most important task. Both metal ions and their reduced forms need to be protected during the reaction, and the semi-qualitative theory of hard/soft acid/base (HSAB) can be a useful guide for the selection of different stabilizing agents. Metal ions and atoms are both Lewis acids, and a strong Lewis acid–base interaction can be achieved by using suitable Lewis bases according to the HSAB principle.^36^ Generally, R-NH_2_ (R for alkyl chain) readily coordinates with metal ions, and R-SH is effective in stabilizing noble metals. Consequently, we used a mixture of two different reagents during the synthesis of nanoclusters of noble metals. Table 1 lists representative reagents used in our syntheses. For metals like copper, the amine group can not only coordinate with copper ions^37^ but also attach to the copper surface to direct its growth.^38^ Therefore, triethanolamine alone can be used to prepare Cu–SiO_2_ HHNs.

**Table 1:** Reagents Used for the Syntheses of Different Nanoclusters as Guided by the HSAB Theory

metal ions	ligands for metal ions	ligands for metals
Au^3+^, Pt^4+^	HOCH_2_CH_2_NHCH_2_CH_2_NH_2_	HOCH_2_CH_2_SH
Ag^+^, Pd^2+^	HOCH_2_CH_2_NHCH_2_CH_2_NH_2_	HOCH_2_CH_2_SCH_2_CH_2_OH
Ru^3+^	HOCH_2_CH_2_NHCH_2_CH_2_NH_2_	HOCH_2_CH_2_NHCH_2_CH_2_NH_2_
Co^2+^, Ni^2+^	(CH_2_NH_2_)_2_	(CH_2_NH_2_)_2_
Fe^3+^, Pb^4+^	NaOOCCHOHCHOHCOONa	NaOOCCHOHCHOHCOONa
Cu^2+^	N(CH_2_CH_2_OH)_3_	N(CH_2_CH_2_OH)_3_

**Figure 3:**
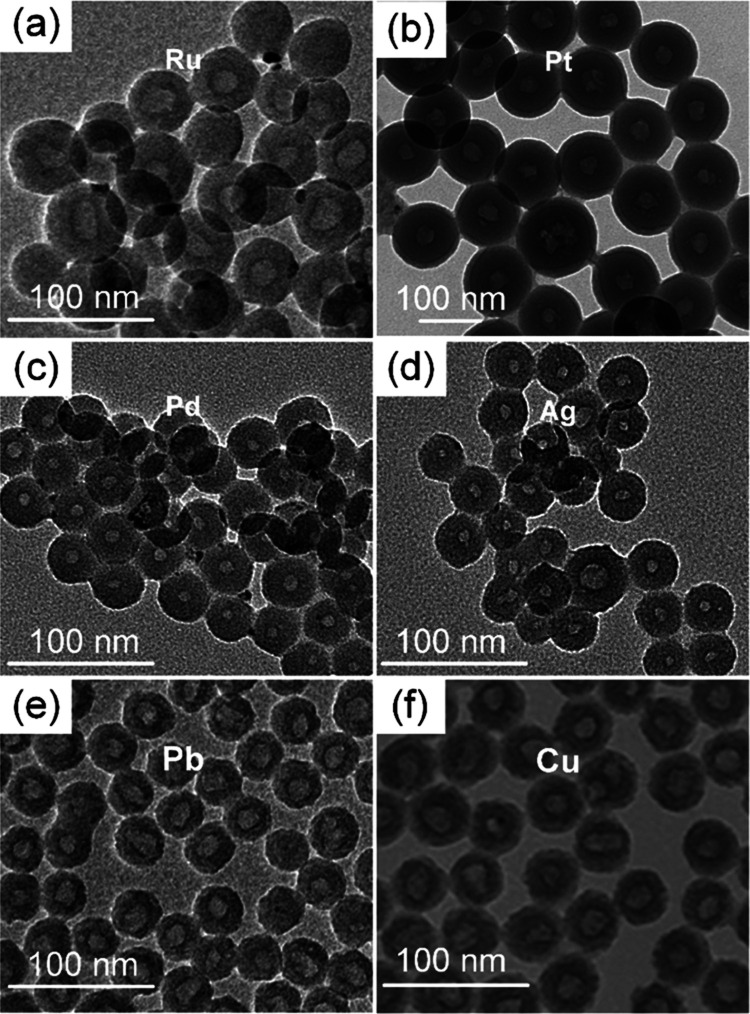
TEM images of HHN samples prepared with different metals. (a) Ru-SiO_2_. (b) Pt–SiO_2_. (c) Pd–SiO_2_. (d) Ag–SiO_2_. (e) Pb–SiO_2_. (f) Cu–SiO_2_.

Figure 3 shows TEM images of different metal nanoclusters synthesized in this way, including Ru, Pt, Pd, Ag, Pb, and Cu. In all cases, metal nanoclusters form with the help of the selected capping agents and are then encapsulated in the silica matrix to form HNN structures. The small size of the embedded metal nanoclusters and the low phase contrast between metal nanoclusters and silica again preclude imaging of these nanoclusters inside the silica matrix. As for Au–SiO_2_, sintering of these small clusters into larger particles reveals their existence inside the silica matrix (see Figure SI8 for Cu–SiO_2_ HHNs as a representative sample). It is noteworthy that a detailed TEM investigation reveals that virtually all of these aggregated crystalline nanoparticles are embedded in the sintered silica matrix and do not show any preferential location with respect to their distribution inside the silica. This suggests that the original metal nanoclusters are similarly evenly distributed across the original silica matrix, i.e., the HHNs have a uniform shell composed of metal nanoclusters and silica.

**Figure 4:**
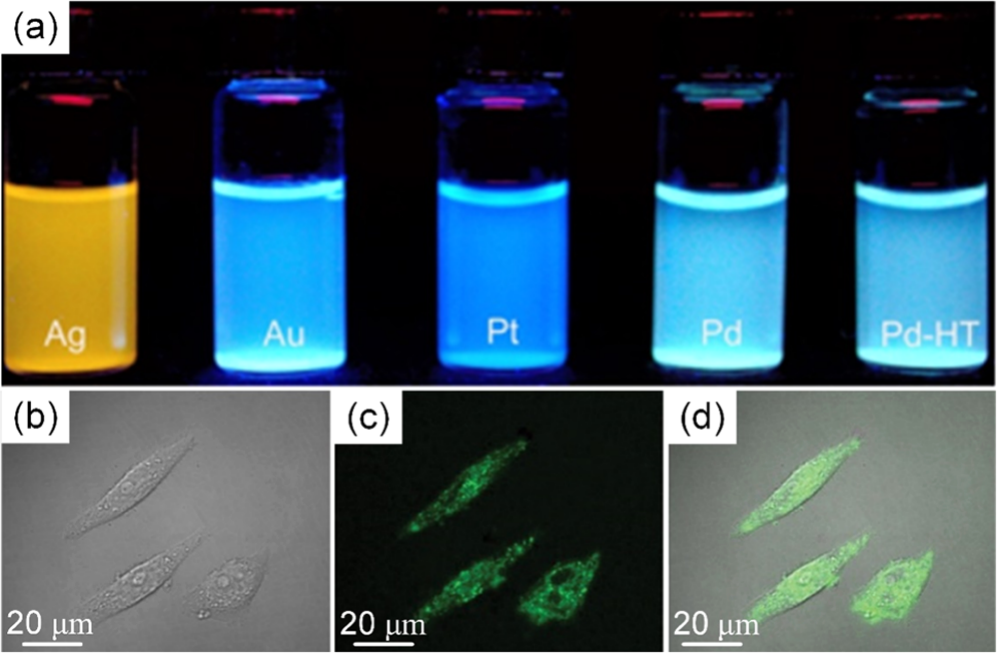
Photos of different HHN suspensions illuminated at 365 nm and images of living MCF-7 cells incubated with 20 μg/mL Pd–SiO_2_ HHNs for 3 h. Top (a): From left to right: Ag–SiO_2_, Au–SiO_2_, Pt–SiO_2_, Pd–SiO_2_, and Pd–SiO_2_ heated at 600 °C for 2 h. Bottom: Bright field image (b), fluorescent image irradiated by a 405 nm laser (c), and overlay of bright field and fluorescent images (d).

Similar to Au, we tested the fluorescence of representative solutions of noble metal HHNs (Figure 4). SiO_2_-stabilized nanoclusters possess two interesting characteristics favorable for their use as robust fluorescent probes. First, a large number of nanoclusters is embedded in each nanosphere, resulting in much stronger fluorescence due to the superposition of their signals. Second, the silica matrix provides an excellent shield for the nanoclusters against harsh environments. For example, the fluorescence of Pd–SiO_2_ HHNs can not only sustain a long period of UV exposure (10 h in our test) but also survive a high-temperature treatment up to 600 °C. As shown in Figure 4a, the sample still shows strong fluorescence after high-temperature treatment, with no obvious decay. To the best of our knowledge, this is the first report of fluorescent nanoclusters with such high thermal stability.

The strong and extremely stable fluorescence of noble metal nanoclusters makes them especially promising as fluorescent probes in biology. Here, we present some preliminary results on detecting MCF-7 (human breast cancer cells, Figure 4b) by using Pd–SiO_2_ HHNs as the light-emitting probe. Due to the good biocompatibility of the SiO_2_ matrix, the Pd–SiO_2_ HHN powder can be easily dispersed in water and then be directly incubated into the cell with no need for further surface modification. As shown in Figure 4c,d, the Pd–SiO_2_ HHNs show strong fluorescence and can illuminate the detailed structure of MCF-7. Compared to commonly used organic fluorescent dyes, the Pd–SiO_2_ HHNs show highly stable performance upon extended UV exposure. Combined with their excellent biocompatibility and low cytotoxicity,^39^ these Pd–SiO_2_ HHNs hence constitute promising cell imaging agents.

**Figure 5:**
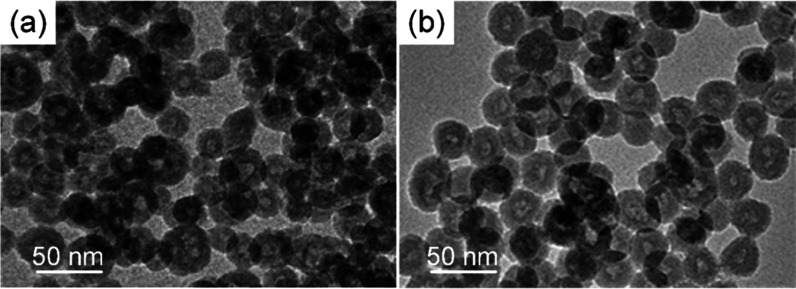
TEM images of HHN samples for different metals. (a) Fe(OH)_3_. (b) (NH_4_)_6_Mo_7_O_24_.

All of the syntheses discussed so far target metal nanoclusters. However, for metals like Fe, it is difficult to maintain metallic character due to their low reduction potential (e.g., −0.04 V for Fe^3+^/Fe vs 1.5 V for Au^3+^/Au), especially when highly reactive nanoclusters are targeted. However, our proposed synthesis mechanism suggests that it is the surface functional groups rather than the nanoclusters themselves that are interacting with the surfactant at the emulsion interface. We hence hypothesized that different kinds of nanoclusters besides metals can similarly be anchored and stabilized at the interface as long as two basic requirements are satisfied: a fast precipitation, which yields very small NPs, and a suitable surface modification. Indeed, we found that Fe^3+^-containing complexes, instead of metallic Fe, can also be prepared in nanocluster form with the help of the emulsion interface. Sodium tartrate was selected as an additive to stabilize Fe^3+^ according to the HSAB principle: the carboxylate group can strongly coordinate with Fe^3+^ and has the necessary hydroxyl group to coordinate with the surfactant at the W/O interface. The ammonium hydroxide used for the hydrolysis of TEOS provides basic conditions (pH ∼ 9.5) that induce Fe^3+^ precipitation to form a brown suspension (*K*_sp_ = 6 × 10^–38^ for Fe(OH)_3_ at 25 °C). Figure 5a shows a typical TEM image for the prepared hydroxide HHN sample. Interestingly, small but discernible nanoclusters can be observed around the central cavity of the hollow silica nanospheres, again suggesting the location of the nanoclusters around the emulsion interface and existence of a Pickering stabilization during the formation of these a nanocapsule structure. The complexed Fe(OH)_3_ will convert to metal oxide during calcination of the formed hollow silica nanospheres. The presence of Fe complexes and oxides in the hollow silica nanospheres was first verified via Raman spectroscopy. The Raman spectra of Fe_2_(C_4_H_4_O_6_)_3_–SiO_2_ (before calcination) and Fe_2_O_3_@SiO_2_ (after calcination) hollow spheres are shown in Figure SI9a,c. The peaks at 1395, 1598, 2331, and 2938 cm^–1^ are characteristic of tartaric acid ions (C_4_H_4_O_6_^2–^), and the peak at 631 cm^–1^ is due to the Fe–O stretch in the complex, confirming the presence of the Fe tartaric acid complex.^40^ After high-temperature treatment (500 °C for 2 h), the C_4_H_4_O_6_^2–^ signals disappear and are replaced by the characteristic peaks of Fe–O (378, 573, and 708 cm^–1^), indicating the formation of iron oxide nanoclusters.^41^ The presence and nature of Fe after calcination is further confirmed via XPS analysis (see Figure SI10a). The typical Fe 2p core-level spectra of the HHN sample indicate the formation of iron oxide during the heat treatment. The two main peaks at 724.7 and 711.3 eV attributed to the Fe 2p_1/2_ and Fe 2p_3/2_ states, respectively, can be further deconvoluted into two peaks, indicating the coexistence of Fe^3+^ (50.5%) and Fe^2+^ (49.5%) and thus confirming the presence of both ferrous and trivalent iron and the absence of metallic Fe in these structures.

Following the same reasoning, the approach can be further transferred even to the preparation of metal salt HHNs by adapting the synthetic protocol. Taking the synthesis of metal-containing polyanions as an example, it is known that different ammonium salts can precipitate polyanions such as [Mo_7_O_24_]_6_.^42^ We find that an organic ammonium salt, such as triethanolamine hydrochloride, effectively stabilizes (NH_4_)_6_Mo_7_O_24_ nanoclusters. (NH_4_)_6_Mo_7_O_24_ first precipitates in the water droplets, and the precipitating clusters are then caught and anchored at the water–oil interface due to the existence of hydroxyl groups in the organic ammonium salt. In this way, the Pickering stabilization effect can also be used to form hollow nanospheres of (NH_4_)_6_Mo_7_O_24_–SiO_2_, as shown in Figure 5b. Raman spectroscopy of the (NH_4_)_6_Mo_7_O_24_–SiO_2_ HHNs (Figure SI9b) confirms the presence of a peak at 937, 890, 365, and 219 cm^–1^, which are characteristic of heptamolybdate species (Mo_7_O_24_^6–^),^43^ confirming that Mo is present in the nanoparticles in the form of Mo_7_O_24_^6–^ and SiO_2_@MoO_3_ hollow spheres. After high-temperature treatment in air (500 °C for 2 h; Figure SI9d), Raman peaks appear at 992, 286, 152, and 120 cm^–1^ and are attributed to the α-MoO_3_ phase, while much sharper peaks at 868, 820, 745, 411, 375, and 338 cm^–1^ could be assigned to the β-MoO_3_ phase.^43^ Further analysis via XPS test confirms the formation of oxide nanoclusters of MoO_3_ during heat treatment (Figure SI10b).

## Conclusions

In summary, we report a highly flexible synthetic protocol for the synthesis of a wide range of very small, silica-embedded nanoclusters, including metals, metal hydroxides, and metal salts. The flexibility of the approach is enabled by detailed insights into the underlying synthesis mechanism, which is based on use of the emulsion interface of a W/O microemulsion as an effective “docking area” for the stabilization of active nanoclusters. This nanocluster enrichment in turn stabilizes the emulsion interface due to a Pickering stabilization effect. During the subsequent sol–gel process, the nanoclusters are encapsulated in situ inside a porous silica matrix, resulting in a hybrid hollow nanostructure (HHN). This unique composite nanostructure endows these metal-silica HHNs with a combination of favorable properties, including high thermal stability, good biocompatibility, and excellent photostability. We expect that these HHNs and their underlying synthesis mechanism will offer new perspectives for materials chemistry, catalysis, optics, and bioimaging.

## Supporting Information Available

Materials, synthetic procedures, and detailed material characterizations (PDF)
