# Supplementary material for: General Method to Synthesize Highly Stable Nanoclusters via Pickering-Stabilized Microemulsions
Source: Langmuir. 2023 Apr 18;39(17):6126–33. doi: 10.1021/acs.langmuir.3c00221 (PMC10157882; doi:10.1021/acs.langmuir.3c00221)
Supplement: Supplementary file 1 — la3c00221_si_001.pdf [file la3c00221_si_001.pdf]

Supporting information

A General Method to Synthesize Highly Stable Nanoclusters via Pickering-Stabilized Microemulsions

Wei Zou[1], Cui Wang[1], Jiasheng Wang[1], Jia Xiang[1], Götz Veser[2,*], Shufen Zhang[1], Rongwen Lu[1,2,*]

1 State Key Laboratory of Fine Chemicals, Frontiers Science Center for Smart Materials Oriented Chemical Engineering, Dalian University of Technology, Dalian 116024, P. R. China.

2 Department of Chemical Engineering, University of Pittsburgh, Pittsburgh, PA 15261, USA

* Corresponding author, E-mail: lurw@dlut.edu.cn, gveser@pitt.edu

Table of Contents

A. Materials and Instrumentation **S-2**

B. Synthetic Procedures **S-3**

C. Figures **S-5**

A Materials and Instrumentation

1. Materials

The chemicals used in this study were of analytical grade and purchased from reputable sources. Brij 58 (GR) and mercaptoethane ($HSCH_2CH_3$, 99%) were obtained from ACROS Organics, while chloroauric acid hydrated ($HAuCl_4 \cdot 4H_2O$), silver nitrate ($AgNO_3$), ferric chloride ($FeCl_3 \cdot 6H_2O$), cobalt nitrate ($Co(NO_3)_2 \cdot 6H_2O$), cupric nitrate ($Cu(NO_3)_2 \cdot 3H_2O$), chloroplatinic acid ($H_2PtCl_6 \cdot 6H_2O$), palladium chloride ($PdCl_2$), ruthenium(III) chloride ($RuCl_3 \cdot 3H_2O$), lead nitrate ($Pb(NO_3)_2$), ammonium molybdate ($(NH_4)_6Mo_7O_{24} \cdot 4H_2O$), ammonium paratungstate ($(NH_4)_6W_7O_{24} \cdot 6H_2O$), mercaptoacetic acid ($HSCH_2COOH$), triethanolamine ($N(CH_2CH_2OH)_3$), ethylenediamine ($NH_2CH_2CH_2NH_2$), sodium tartrate ($Na_2C_4H_4O_6 \cdot 2H_2O$), cyclohexane, isopropanol, and 4-nitrophenol were all purchased from Sinopharm Chemical Reagent Co. Ltd. Similarly, mercaptoethanol ($HSCH_2CH_2OH$, CP), sodium borohydride ($NaBH_4$, 96%), hydrogen peroxide (H_2O_2, 30 wt%), hydrazine hydrate ($N_2H_4 \cdot H_2O$, 80 wt%), ammonium hydroxide (NH_4OH, 28 wt%), and hydrochloric acid (HCl, 35 wt%) were also purchased from Sinopharm Chemical Reagent Co. Ltd. N-(2-hydroxyethyl)ethylenediamine ($HOCH_2CH_2NHCH_2CH_2NH_2$, 99%) was procured from Alfa Aesar, while dihydroxyethylsulphide ($HOCH_2CH_2SCH_2CH_2OH$) was obtained from Strem Chemicals Inc. All chemicals were used without further purification. The water used in all experiments was prepared by passing through an ultra-pure purification system. MCF-7 cells were provided by Prof. Jingyun Wang of the School of Life Science and Biotechnology at Dalian University of Technology.

2. Instrumentation

Scanning electron microscopy (SEM) was performed using a Hitachi S-5500 SEM equipped with a scanning transmission electron microscopy (STEM) mode. Transmission electron microscopy (TEM) images were captured with a FEI Tecnai G^2 20 microscope operating at 200 kV. X-ray photoelectron spectroscopy (XPS) was carried out using an EscaLab 250 X-ray photon-electron spectrometer (Thermo VG Scientific). X-ray diffraction (XRD) measurements were conducted with a RIGAKU D/MAX-2400 diffractometer. Nitrogen adsorption isotherms of the Au-SiO2 hollow hybrid nanoparticles (HHNs) were obtained with an ASAP 2020 adsorption analyzer (Micromeritics) at liquid nitrogen temperature. UV-visible spectra were acquired using an Agilent 8453 UV–visible diode array spectrophotometer. Photoemission spectra were obtained with a FP-

6500 spectrofluorometer (JASCO). Dynamic light scattering (DLS) analysis of microemulsion was performed at 50 °C using a ZETASIZER Nano-ZS90 dynamic light scattering system (Malvern instruments).

Fourier transform infrared spectroscopy (FT-IR) using a Mettler-Toledo ReactIR 15 spectrometer with diamond attenuated total reflectance (ATR) probe was employed to investigate the molecular interaction between Brij 58 and mercaptoethanol. Spectra were acquired at 50 °C with a resolution of 2 cm^{-1} in the wavenumber range of 900 – 1400 cm^{-1}, with cyclohexane spectrum used as the background. FT-IR spectra of the stretching vibration of C-O-C in mercaptoethanol Brij 58 cyclohexane solution were obtained with varying amounts of mercaptoethanol from 0, 0.45, 0.75, 1.05, and 1.35 mL. Spectral shifting in the C-O-C peak revealed the interaction of mercaptoethanol with Brij 58.

For the in vitro imaging of Pd-SiO2 HHNs, 20 μL of 1 g/L Pd-SiO_2 HHNs was added to MCF-7 human breast cancer cells (5×10^5 cells in 1 mL of DMEM) grown in a glass-bottom culture dish and incubated at 37 °C (5% CO_2) for 3 h. The cells were rinsed in phosphate-buffered saline (PBS) buffer three times to remove external nanoparticles. The glass-bottom culture dish was then filled with cell-growth medium and the Pd-SiO_2 HHNs-tagged cells were imaged with an Olympus FV1000-IX81 confocal fluorescence microscope using 405 nm excitation. The signals were detected at wavelengths ranging from 415 to 480 nm, and the cell morphology was imaged at the same time and merged automatically with the fluorescent image.

B. Synthetic Procedures

1. Synthesis of Au-SiO_2 HHNs:

10 g of Brij 58 was dissolved in 50 mL of cyclohexane at 50 °C, resulting in a clear solution. A mixture of 1.5 mL of 0.5 M aqueous $HAuCl_4$ solution and 0.5 mL of N-(2-hydroxyethyl) ethylenediamine was added dropwise to the formed microemulsion, followed by the addition of 0.5 g of $NaBH_4$ powder to reduce $HAuCl_4$ to Au nanoparticles. The solution underwent a color change from orange to dark red. Next, 0.4 mL of mercaptoethanol was added to the solution, which continued to react for 2 h. To this solution, 4.0 mL of 28 wt% ammonium hydroxide was added and stirred for 20 min. Finally, 6 g of TEOS was added slowly and continuously, and the sol-gel process was allowed to proceed for 2 h at 50 °C. The resulting solid sample was precipitated out by adding

a large amount of isopropanol. After three cycles of washing and centrifugation with a mixture of isopropanol and water (3:1), the Au-SiO_2 HHNs were collected, dried overnight, and calcined at 400 °C for 2 h to remove any residual surfactant. A typical synthesis yielded several hundred grams of nanomaterials.

2. Synthesis of noble M-SiO_2 HHNs:

For the synthesis of other noble metal-SiO_2 HHNs such as Ag, Pd, and Pt, a similar procedure to that of Au was employed, except for the use of different ligands for the metal ions. Notably, Pd was treated in an H_2 environment at 400 °C for 2 h to prevent oxidation to PdO.

3. Synthesis of non-noble M-SiO_2 HHNs:

For the synthesis of non-noble metal-SiO_2 HHNs such as Cu, Co, Ni, Pb, Ru, the stabilization agents for both metal ions and reduced metals were the same as listed in Table 1.

For the synthesis of Fe-SiO_2 HHNs, no reducing agent was necessary, and sodium tartrate was used as the stabilizing agent. A mixture of 1.5 mL of 1.0 M Fe^{3+}-complex solution (prepared by dissolving 2.7 g $FeCl_3·6H_2O$ and 3.5 g $Na_2C_4H_4O_6·2H_2O$ in 10 mL water) was added to the microemulsion system, resulting in a yellow-colored homogeneous microemulsion. Subsequently, 3.0 mL of 28 wt% ammonium hydroxide solution was added to the microemulsion to form a brown suspension. Finally, TEOS was introduced slowly to form the HHNs structure.

4. Synthesis of M_O-SiO_2 HHNs:

For the synthesis of Mo and W-SiO_2 HHNs, triethanolamine hydrochloride was used as the stabilizing agent. Initially, a 0.1 M aqueous solution of metal salt, using ammonium molybdate as an illustration, was prepared by the aid of hydrogen peroxide. Subsequently, 1.5 mL of the freshly prepared yellow solution was added to the oil phase, along with 1.2 mL of 1 M triethanolamine hydrochloride. Hydrazine hydrate was utilized to react with the hydrogen peroxide, leading to the formation of a suspension of ammonium molybdate due to the consumption of hydrogen peroxide. Finally, 4.0 mL of 28 wt% ammonium hydroxide solution was added, and the subsequent steps were identical to those for the synthesis of Au-SiO_2.

C. Figures

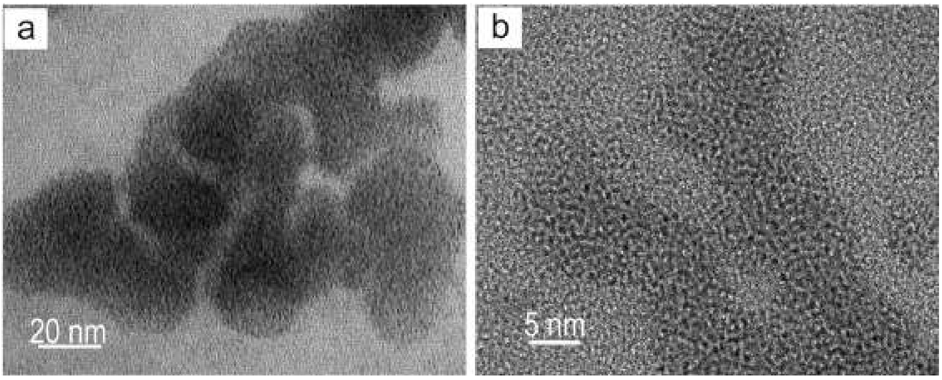

Figure SI1 TEM images of as-synthesized Au nanoclusters prepared in microemulsion. a, TEM. **b**, High-resolution TEM. Due to the absence of a stabilizing shell in the absence of TEOS, the Au nanoclusters form loose, large aggregates (the instability of the small nanoclusters under the electron beam renders them difficult to discern in the final TEM image).

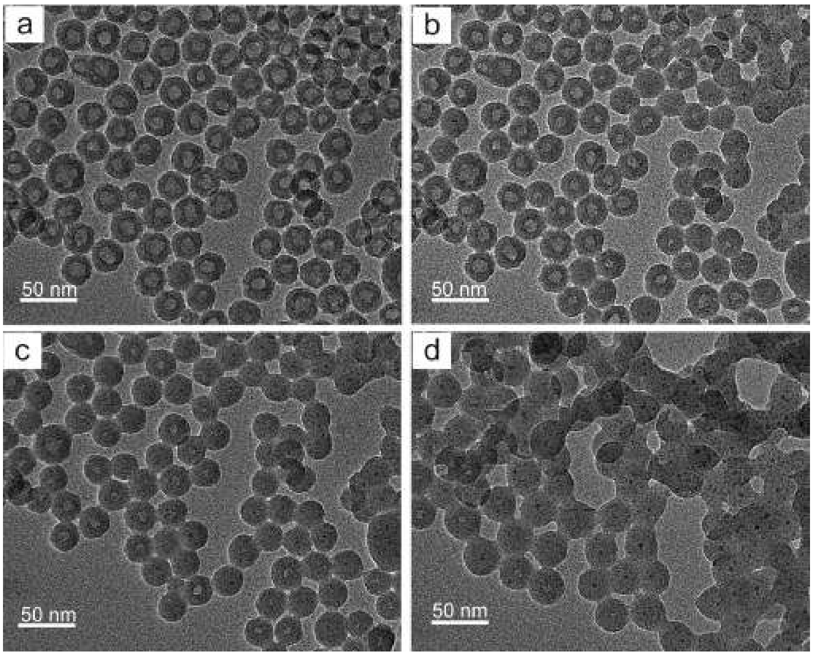

Figure SI2 TEM images of the hollow nanospheres. a-d, Nanospheres collected vs time in TEM: 0 sec (**a**), 30 sec(**b**), 60 sec (**c**), and 90 sec (**d**). Rapid collapse of the nanostructure under the incident electron beam is observed.

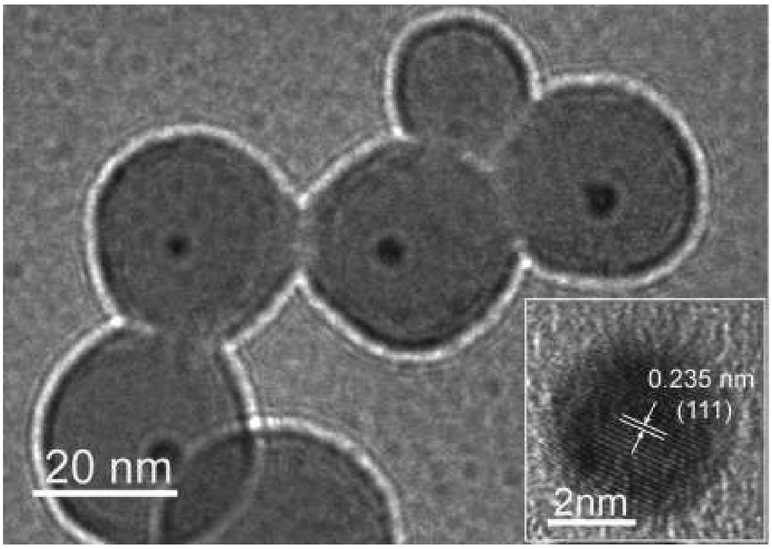

Figure SI3 TEM image of Au-SiO_2 HHNs after heating to 600 ^oC. (Inset) High-resolution TEM image shows the lattice planes separated by about 0.235 nm, corresponding to the (111) lattice spacing of the face-centered cubic Au.

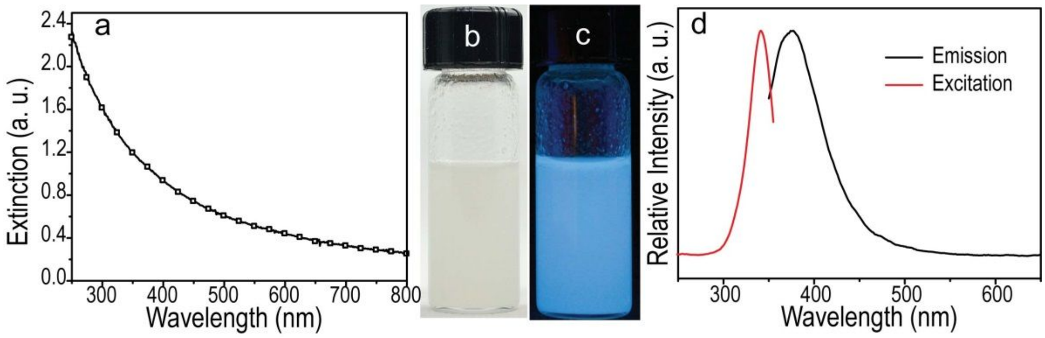

Figure SI4 Optical spectra and photos of Au-SiO_2 HHNs samples. a, d, UV-vis extinction spectrum (**a**) and fluorescent spectrum (**d**) of aqueous suspensions of Au-SiO_2 HHNs. **b, c,** Photos taken under visible (**b**) and UV light (365 nm) (**c**).

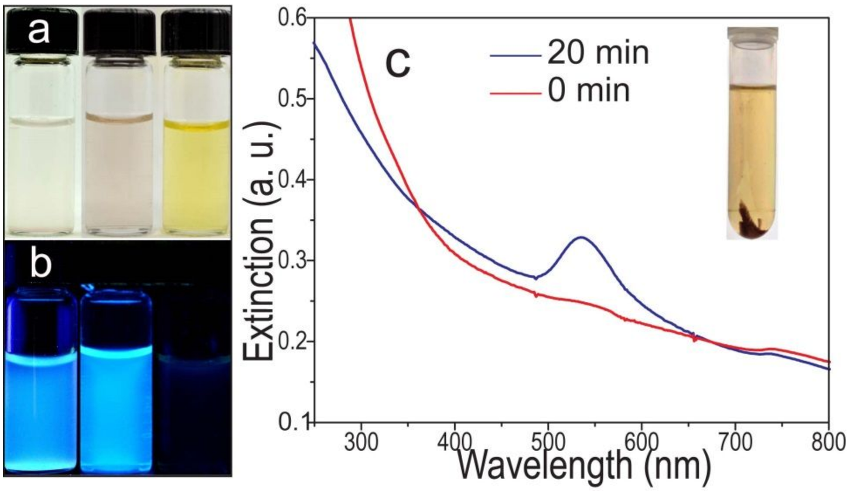

Figure SI5 Photos and optical spectra of suspensions of Au-SiO_2 HHNs samples after different treatments. a, From left to right: pristine sample, HF-etched sample, and aqua regia-treated sample. **b**, corresponding fluorescent images of these three samples. **c**, UV-vis extinction spectra of the Au-SiO_2 HHNs samples collected at 0 min (red line) and 20 min (blue line) after the introduction of HF. The inset shows the centrifuged sample after a 20 min treatment of HF etching.

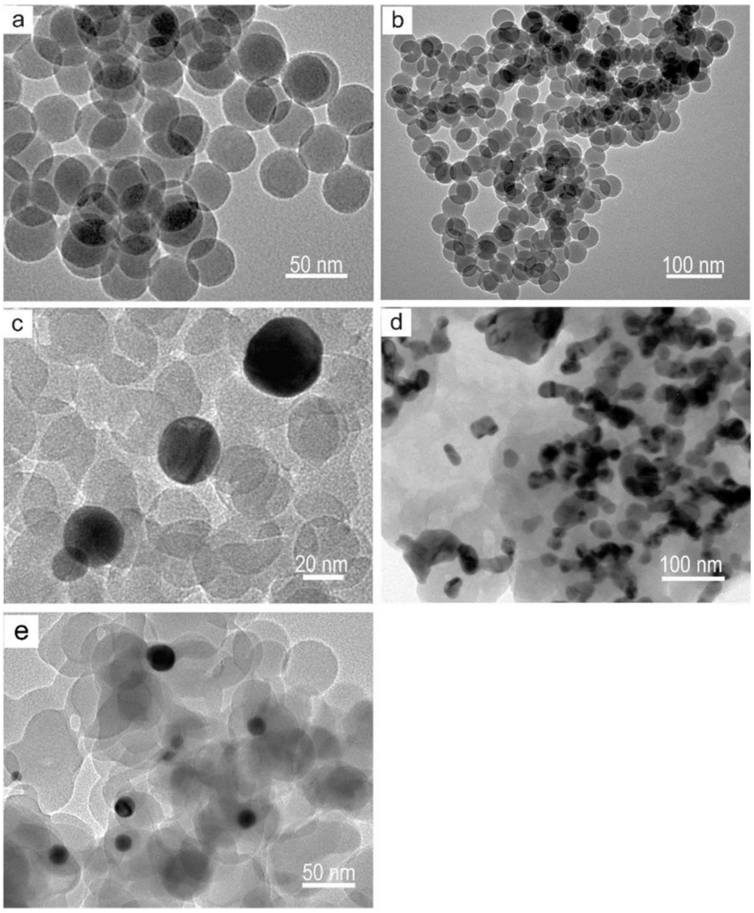

Figure SI6 TEM images of samples from control experiments. a-c, one reactant is selectively

excluded for $HAuCl_4$ (**a**), $NaBH_4$ (**b**) and mercaptoethanol (**c**). **d-e**, mercaptoethanol is replaced by ethanolamine (**d**), and mercaptoethane (**e**).

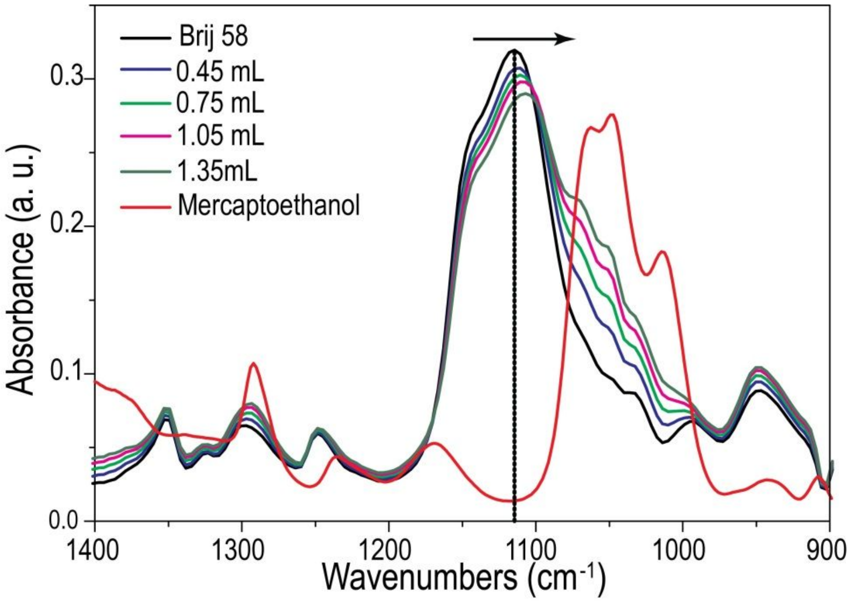

Figure SI7 FT-IR spectra of a mixed solution composed of Brij 58, cyclohexane and mercaptoethanol. The $\nu_{C\text{-}O\text{-}C}$ peak originated from Brij 58 gradually shifts to lower wavenumbers with a continuous increase of the amount of mercaptoethanol.

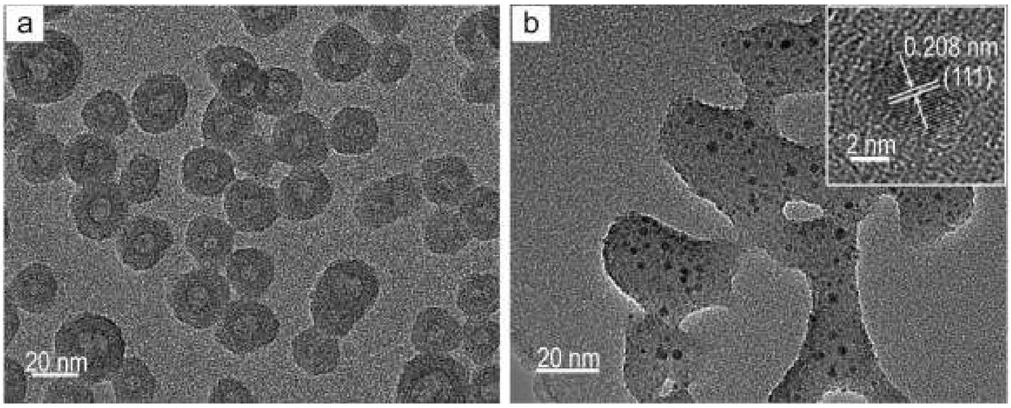

Figure SI8 TEM images of Cu-SiO_2 HHNs. **a**, Before the heat treatment. **b**, After the heat treatment at 600 °C. The inset in (**b**) shows the HRTEM image of a selected copper nanoparticle with its (111) crystalline plane revealed.

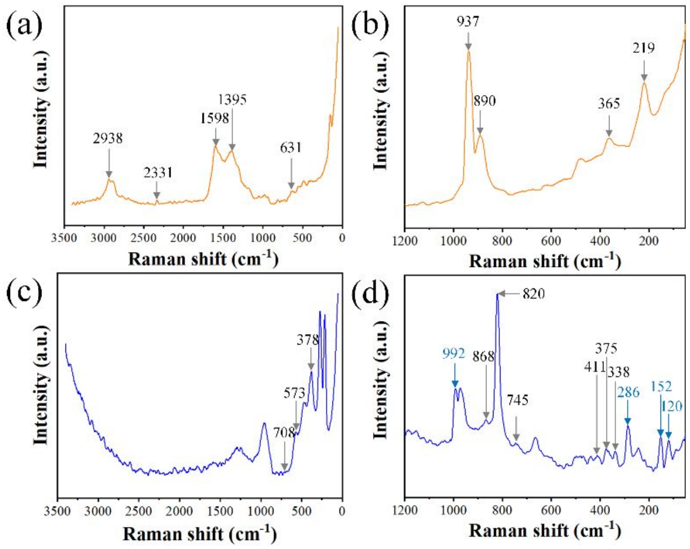

Figure SI9 Raman spectra of (a) $Fe_2(C_4H_4O_6)_3$-SiO_2, (b) $(NH_4)_6Mo_7O_{24}$-SiO_2, (c) $SiO_2@Fe_2O_3$, and (d) $SiO_2@MoO_3$

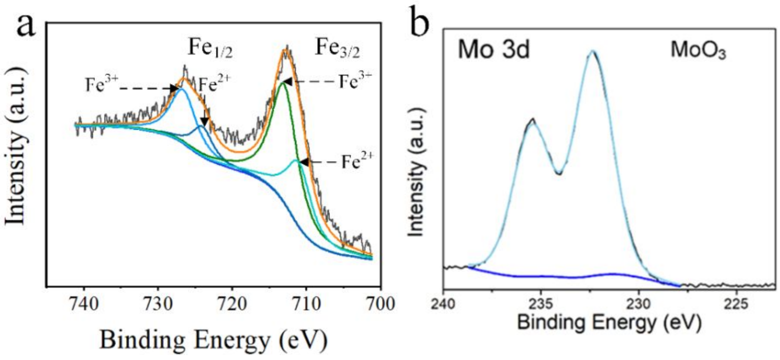

Figure SI10 XPS spectra of HHNs samples. **a**, Fe_2O_3. **b**, MoO_3.
